# Supplementary material for: Patterns of Intron Gain and Loss in Fungi
Source: PLoS Biol. 2004 Nov 30;2(12):e422. doi: 10.1371/journal.pbio.0020422 (PMC532390; doi:10.1371/journal.pbio.0020422)
Supplement: Table S1 — Also available at http://genes.mit.edu/NielsenEtAl/. (4.3 MB ZIP). [file pbio.0020422.st001.zip › NielsenEtAl/html/635.html]

AN5954.1.NCU06279.1.MG05266.1.FG02571.1


```
 CLUSTAL W (1.82) Multiple Sequence Alignments - Introns Inserted


Sequence 1: NCU06279.1	474 aa
Sequence 2: FG02571.1	476 aa
Sequence 3: MG05266.1	471 aa
Sequence 4: AN5954.1	476 aa
Alignment Length: 478 aa
Number Identitical Residues: 324 aa
Alignment Score (without introns) 14666


MG05266.1 	MSGYQNGGP--RGLADDSDVEEEALVADYREQVQYEEGMD----DPDMGLAQQTDDIQSR
NCU06279.1	MSAFQQAQAPARAIDPDSDVEEEALVNDYKEQVQYEEDD---AESTQMSLAAQTDDIQSR
FG02571.1 	MATYQNGVAPARAMDDDSDVEEEALVAEYKEQVQYEDGDDL-SRTTSLNLAQQTDDIQSR
AN5954.1  	-MSYDERANAHPNLNDESDVEEEALVNDYREQVNFDDGMSELDRTTSLGAASQTQDLQAQ
          	   :::   .   :  :********* :*:***::::. .     ..:. * **:*:*::

MG05266.1 	LVAAAQPLDFSAPLEVKFQSYDQYCSLFHFILNSDGPVDLEPPS0YYWAWDVIDEFIYQF
NCU06279.1	LAAAAQPLDYSAGLEVKFSSYDSYCSLFHFILNSEGPVDLEPPS0YYWAWDVIDEFIYQF
FG02571.1 	LVQAAQPLDFQAPLEAKFQSYDAYCSLFHFILNSEGPVDLEPPS0YYWAWDVIDEFIYQF
AN5954.1  	LAAAATPLEYQATLETKFASYDNYCSLFHYILNSDGPVELEVPS0YYWAWDVIDEFIYQF
          	*. ** **::.* **.** *** ******:****:***:** ** ***************

MG05266.1 	NSFSSYRMRIARQANNEEEAQILRENPNTWGCYSVLNVLYSLIQRSQILEQLQAMKRNED
NCU06279.1	NSFSSYRARIARQGNNEEEIAMLRENPNTWGCYSVLNVLYSLIQKSQITEQLAAMKRNED
FG02571.1 	NSFSSYRMRIARQTTNEEERQALRENPNTWGCYSVLNVLYSLIQRSQITEQLGAMKRGED
AN5954.1  	ESFCRYRNRVARSGSNEEEAQLLRENPNTWGCYSVLNVLYSLIQKSQINEQLAAMKRGED
          	:**. ** *:**. .****   **********************:*** *** ****.**

MG05266.1 	PMAVAGDYGSRSLYRMLGYFSIIGLLRVHCLLGDFSLALRTLDDIELNKKAMFARVMAAH
NCU06279.1	PAAVAGEYGSKNLYKMLGYFSIIGLLRVHTLLGDFSLALKTLDDIELNKKAMFARVMAAH
FG02571.1 	PAAVAGEYGNKNLYKMLGYFSIIGLLRVHCLLGDFSLALKTLDDIELNKKAMFARVMAAH
AN5954.1  	PAAFAGEYGSRPLYKMLGYFSIIGLLRVHCLLGDFTLALKTLDDIEMNKKAMFARVMAAH
          	* *.**:**.: **:************** *****:***:******:*************

MG05266.1 	FTTYYYVGFSYMMVRRYADAIRMFSHILVYVSRTKNFQKN-AQYDSITKKNDQMLALIAI
NCU06279.1	FTTYYYVGFSYMMMRRYADAIRMFSHILVYVSRTKNFQKN-AQYDSITKKNDQMYALIAI
FG02571.1 	FTTYYYVGFSYMMQHRYADAIRMFSHILIYVSRTKNFQKS-AQYDSITKKNEQMYALIAI
AN5954.1  	FTTYYYVGFSYMMTRRYGDAIRMFSHILVYVSRTKNFQKGGNSYDAIAKKNDQMYALIAI
          	************* :**.**********:**********.. .**:*:***:** *****

MG05266.1 	CVAFHPTRLDDTIHTALREKFGDQLLKLQRGGPESLPVYEELFRTACPKFISPVPPDFDN
NCU06279.1	CVAFQPTRLDDTIHTALREKYGDQLLKLQRGGPEALPIYEELFRTACPKFISPVPPNFDE
FG02571.1 	CVAFQPTRLDDTIHSALREKYGDQLLKLQRGGPESLPIFEELFRAACPKFISPVPPDFEN
AN5954.1  	CVALHPTRLDDTIHSALREKYGEQLHRLQQGGPEALPLFEELFRSACPKFISPTPPDFDN
          	***::*********:*****:*:** :**:****:**::*****:********.**:*::

MG05266.1 	PEANVDPIEHHLSVFMDEVKTNMWSPTVKSYLRLYTTMDLKKLAGFLSVKPEELRSWLLV
NCU06279.1	PEANIDPIEHHLSVFMDEVKTNMFNPTIKSYLRLYTTMDLKKLAGFLEVKPEELRGWLMV
FG02571.1 	PEANIDPVEHHLAVFMDEVKTNMWSPTIKSYLRLYTTMDLNKLAGFLEVKPEELRSWLLV
AN5954.1  	PALNVDPVDHHTAIFMDEVKNTLYNPTIRSYLKLYTTMDLQKLAGFLDVEPEKLRSWLLV
          	*  *:**::** ::******..::.**::***:*******:******.*:**:**.**:*

MG05266.1 	NKQRTKQLRWADHGLLDGELVNVSDLDYAMQG0DLIHISEAKVGRKLVDWYLRNLSRTYV
NCU06279.1	NKQRTKQLRWTDGGLLEGELVNVSDLDYALQG0DLIHISEAKVGRKLVDWYLRNLSRTYA
FG02571.1 	TKQRTKQLRWQDQGLLDGELVNVSDLDYALQG0DLIHISEAKVGRKLVDWYLRNLSRTYN
AN5954.1  	NKQRSRQVRWVEGGLLEGETVSANDLDYALEK~DLIHVSETKAGRRLVDWYLRNLARVY-
          	.***::*:** : ***:** *...*****::  ****:**:*.**:*********:*.* 

NCU06279.1	
FG02571.1 	
MG05266.1 	
AN5954.1  	
          	
```
